# Host–Microbiota Interactions in the Pathogenesis of Porcine Fetal Mummification

**DOI:** 10.3390/microorganisms13051052

**Published:** 2025-04-30

**Authors:** Mingyu Wang, Lin Zhang, Zhe Liu, Ao Guo, Gongshe Yang, Taiyong Yu

**Affiliations:** Key Laboratory of Animal Genetics, Breeding and Reproduction of Shaanxi Province, Laboratory of Animal Fat Deposition & Muscle Development, College of Animal Science and Technology, Northwest A&F University, Xianyang 712100, China

**Keywords:** pigs, gut microbiota, MUM, mGWAS, multiple omics

## Abstract

The number of mummies (MUM) in pigs is a major factor affecting sow reproductive performance. Reducing the incidence of MUM can effectively improve sow utilization efficiency. However, the complex mechanisms by which the host genome, gut microbiome, and metabolome interact to influence sow MUM remain unclear. Based on the current research landscape, this study systematically reveals the regulatory mechanisms of the host genome–gut microbiome-metabolome interaction network on sow MUM. By conducting a multi-omics analysis on the intestinal contents of Yorkshire sows during late gestation across different parities, we constructed a dynamic atlas of the gut microbiota and identified 385 core microbial taxa. Through multi-model MWAS and meta-analysis, we screened six key microbial taxa significantly associated with MUM, including *Bacteroidales_RF16_group*, *Prevotellaceae_Ga6A1_group*, *Comamonas*, *Paraprevotella*, *Dorea*, and *Gallicola*. An mGWAS analysis further identified *Bacteroidales_RF16_group* as regulated by host genetics, as well as candidate genes such as *EGF*, *ENPEP*, and *CASP6*, and important SNP loci such as *rs345237235* and *rs3475666995*. The study found that the abundance of Proteobacteria in the sow’s gut increased progressively from the first parity, providing a theoretical basis for pathogen suppression mechanisms. By integrating fecal metabolomics data, we constructed a four-dimensional regulatory network of host gene–gut microbiota–metabolite–host phenotype. This study innovatively combines quantitative genetics with multi-omics approaches, not only providing a theoretical foundation for understanding host–microbiota interaction mechanisms but also offering critical scientific guidance for reducing sow MUM incidence and improving reproductive efficiency.

## 1. Introduction

In recent years, with the innovation and widespread adoption of high-throughput sequencing technologies, research on host-associated microbiomes has achieved groundbreaking progress [1,2]. These technological advancements have enabled researchers to deeply analyze the compositional characteristics, spatial structure, and dynamic changes of microbial communities, systematically elucidating their complex associations with host phenotypes [3]. Numerous studies have confirmed that the gut microbiome exhibits significant environmental plasticity, with dietary structure and antibiotic use being key external factors influencing its composition [4]. Building on this, the scientific community has gradually established an interaction framework of “host genotype-microbiome-phenotype”. The host genotype shapes the structural characteristics of the microbiome, while the microbiome, in turn, influences host phenotypes through metabolic regulation and other pathways. This theory highlights the importance of in-depth research into host–microbe interactions for understanding their roles in health maintenance, disease development, and individual growth [4,5]. At the molecular level, studies have found that specific microbes can form biofilm barriers through specific adhesion to the intestinal mucosa. For example, *Lactobacillus* bacteria bind specifically to intestinal epithelial cells via their surface S-layer proteins [6], not only strengthening the intestinal barrier function but also effectively inhibiting pathogen colonization and invasion. Notably, the surface layer protein A of *Lactobacillus acidophilus* can target colorectal cancer cells, significantly enhancing intercellular connections and reducing epithelial permeability by inducing the expression of the tight junction protein ZO-1 [7,8]. Additionally, gut microbes exert important physiological regulatory effects through their metabolites. By producing short-chain fatty acids, organic acids, and other metabolites, they lower intestinal pH, promote peristalsis, and enhance the competitive advantage of probiotics, thereby inhibiting pathogen colonization. The gut microbiota maintains host health through multiple mechanisms. For instance, Bifidobacteria secrete teichoic acids and glycosidases to inhibit pathogen colonization; Lactobacilli produce bacteriocins, enhancing antimicrobial effects. Additionally, microbial metabolites can stimulate host immunity, collectively sustaining intestinal microecological balance and health [9]. Therefore, research on the gut microbiota helps researchers better understand various physiological changes during animal growth, revealing the mechanisms underlying the occurrence and development of physiological diseases and providing a reliable scientific basis for the targeted regulation of host physiological states.

As a vital source of high-quality protein in the human diet, the pig industry plays a pivotal role in the global food supply chain. Confronted with the dual challenges of a growing global population and rising demand for animal protein, achieving sustainable development and enhancing production efficiency in pig farming have emerged as critical priorities for the livestock industry [10,11]. Within the economic evaluation framework of pig farming, reproductive performance stands as a core metric, driving researchers to explore advanced multi-omics integration technologies to improve sow reproductive efficiency. The occurrence of mummified fetuses in sows is a complex quantitative trait influenced by multiple minor-effect genes and modulated by genetic background, disease infections, nutritional status, and environmental factors. With a heritability of approximately 0.10, traditional breeding methods have yielded limited success in improving this trait [12]. Consequently, identifying key genomic loci and functional genes that regulate mummified fetus numbers is essential for accelerating the genetic enhancement of sow reproductive performance. Recent advances in genome-wide association studies (GWAS) have identified several candidate genes significantly associated with stillbirth rates and mummified fetus numbers, including *BMPER*, *HMGAI*, and *GRAMDIB* [12,13,14]. Nevertheless, a comprehensive understanding of the molecular genetic mechanisms underlying mummified fetus formation remains elusive, highlighting the need for further research. The rapid advancement of biological omics technologies has opened new avenues for elucidating the molecular mechanisms of sow reproductive traits through the integration of genomics, microbiomics, and metabolomics. This multi-omics approach not only unravels the complex regulatory networks governing mummified fetus formation but also enables the precise identification of key genetic loci, offering a robust theoretical foundation for functional validation and breeding applications. Therefore, leveraging multi-omics integration strategies to dissect the molecular genetic basis of mummified fetus numbers in sows and systematically uncover the key regulatory loci and functional genes will be a central focus of future research in this field.

As one of the key indicators directly affecting sow reproductive efficiency, the incidence of mummified fetuses has not yet received sufficient attention in breeding practices. Research indicates that mummified fetuses primarily occur during the critical period from 35 days of gestation to parturition, with an average incidence rate of approximately 5% in Large White and Landrace pig populations. Notably, sow parity structure and nutritional status have been identified as significant risk factors influencing the occurrence of mummified fetuses [15]. To gain a deeper understanding of the dynamic changes in the gut microbiota of sows across different parities and to identify the optimal intervention timing for probiotic supplementation or pathogen suppression, thereby effectively reducing the incidence of mummified fetuses, conducting relevant research holds substantial practical significance. This study systematically analyzed the correlation between the dynamic changes in gut microbiota and the incidence of mummified fetuses in 393 Yorkshire sows during late gestation across different parities. The research not only revealed the proliferation patterns of pathogenic bacteria in the guts of sows of varying parities but also successfully identified the core gut microbiota characteristics of Yorkshire sows. By integrating microbiome-wide association studies (MWAS) and metagenome-wide association studies (mGWAS), we identified the microbial taxa significantly associated with mummified fetuses and their host genetic regulatory networks. To further elucidate the interaction mechanisms between the gut microbiota and host genes, this study innovatively adopted a multi-omics integration analysis strategy, including genomics, metabolomics, and 16S rRNA sequencing technologies. This comprehensive research approach provides new insights into the molecular mechanisms by which host genes regulate the gut microbiota and subsequently influence the formation of mummified fetuses.

## 2. Materials and Methods

All the animal studies were approved by the Institutional Animal Care and Use Committee of Northwest A&F University (approval number: IACUC2024-0823). All operations were carried out in accordance with the university guidelines for animal research. All pigs were cared for and sacrificed following the guidelines of the Institutional Animal Care and Use Committee of Northwest A&F University (Yangling, China).

### 2.1. Animals and Phenotyping

**Figure 1:**
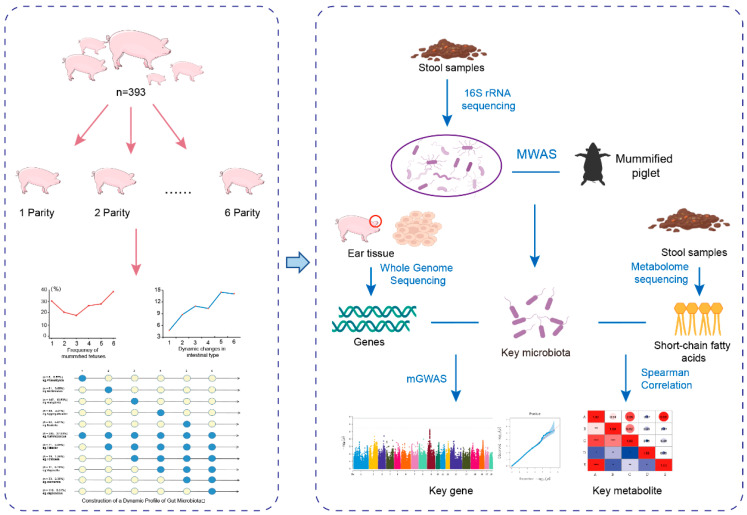
Analysis workflow of the present study.

This study included a total of 393 Yorkshire sows, which were divided into six experimental groups based on their parity (from 1st to 6th parity). All individuals underwent 16S rRNA sequencing, whole-genome sequencing, and phenotypic evaluation. During the experiment, all sows were uniformly managed using the Osborne automated feeding system. All experimental animals were housed in gestation stalls. The dimensions of the sow stalls typically measured 2.0–2.2 m (length) × 0.6–0.7 m (width), allowing the sows to stand and lie down but restricting turning movements to minimize aggression. The flooring primarily consisted of slatted floors to facilitate the drainage of feces and urine into underlying manure channels. The stall structures were constructed with reinforced concrete, standing approximately 1.0–1.1 m in height, with feed troughs and waterers installed at the front. The housing facility was fully enclosed, featuring insulated roofing materials and a mechanical ventilation system to maintain optimal temperature, humidity, and low ammonia concentrations. The sows were housed in different units within the same barn, which was equipped with temperature and humidity control facilities, such as water curtains, to maintain an ambient temperature of 20–23 °C and a relative humidity of 65–80%. All experimental animals were fed a standardized diet consisting of corn and soybean (details are provided in Supplementary Table S1). A statistical analysis of the experimental data was performed using SPSS 22.0 software, with necessary corrections applied to the data. Finally, we performed a statistical analysis on the reproductive phenotypes of all 393 sows and constructed the dynamic changes in the mummified fetus count across parities. The specific experimental design framework and research workflow are detailed in Figure 1.

### 2.2. Sample Collection

In this study, intestinal content samples were collected daily from all 393 experimental sows between 10:00 and 11:00 a.m. post-feeding. Two samples were obtained from each sow and preserved in 2 mL cryotubes for subsequent gut microbiome analysis. Based on breeding records, the parity distribution of the sows was as follows: Primiparous sows (1st parity): 23, 2nd parity: 34, 3rd parity: 67, 4th parity: 70, 5th parity: 101, and 6th parity: 98. The specific operation method is as follows. Rectal palpation was performed using sterile, single-use nitrile examination gloves, and rectal content samples were collected from each animal. Immediately after collection, the samples were transferred into sterile cryotubes and rapidly frozen by immersion in liquid nitrogen. All samples were promptly transported to the laboratory and stored in a −80 °C ultra-low temperature freezer for subsequent analysis.

### 2.3. DNA Extraction and Polymerase Chain Reaction (PCR) Amplification

Microbial DNA was extracted from the fecal samples using the OMEGA Soil DNA Kit (OMEGA Bio-Tek, Norcross, GA, USA) and stored at −20 °C until further analysis. The V3–V4 hypervariable region of the 16S rRNA gene was amplified from the extracted DNA using specific primers, namely the forward primer (5′-ACTCCTACGGGAGGCAGCA-3′) and the reverse primer (5′-GGACTACHVGGGTWTCTAAT-3′), both of which were appended with the Illumina adapter sequence at the 5′ end. The PCR amplification was performed under the following conditions: initial denaturation at 95 °C for 5 min, followed by 30 cycles of denaturation at 95 °C for 30 s, annealing at 55 °C for 30 s, and extension at 72 °C for 30 s, with a final extension at 72 °C for 7 min.

### 2.4. 16S rRNA Gene Sequence Assembly and Clustering

The 16S rRNA sequence data were processed using the QIIME2 2019.4 platform [16]. Sequences were quality-filtered, denoised, and merged using the DADA2 plugin to remove low-quality reads and chimeras. Amplicon sequence variants (ASVs) were subsequently aligned with the MAFFT v7.520 algorithm for accurate phylogenetic placement. Taxonomic classification of the sequences was performed using the Sklearn algorithm within QIIME2, with default parameters, and referenced against the Greengenes database (version 13.8; http://greengenes.secondgenome.com/) accessed on 15 January 2025. 

### 2.5. Bioinformatics and Statistical Analysis

Statistical comparisons of taxonomic abundance were conducted at both the phylum and genus levels across the experimental groups. Alpha and beta diversity indices were analyzed and visualized at the amplicon sequence variant (ASV) level using various methods. Alpha diversity metrics, including the observed species count and the Shannon diversity index, were calculated from the ASV table within QIIME2 and subsequently visualized as boxplots to assess the microbial diversity within each group.

### 2.6. Genotype Data Acquisition and Quality Control

Genomic DNA was extracted from ear tissue samples of all 393 individuals using the Tiangen DNA Extraction Kit (Tiangen Biotech, Beijing, China), strictly following the manufacturer’s protocol. The concentration and purity of the extracted DNA were measured using a Nanodrop instrument (Thermo Fisher Scientific, Waltham, MA, USA), and the DNA integrity was assessed by 1% agarose gel electrophoresis to confirm successful extraction. The DNA was fragmented by sonication, and the resulting fragments were subjected to end repair, dA-tailing, and ligation to Illumina paired-end adaptors. Libraries were constructed by PCR amplification with 500 bp inserts. After amplification and purification, sequencing was performed on the Illumina HiSeq 2500 platform (Illumina, Inc., San Diego, CA, USA) to generate 150 bp paired-end reads. Data quality was ensured by filtering out low-quality reads using FastQC software (http://www.bioinformatics.babraham.ac.uk/projects/fastqc/, accessed on 22 January 2025), with the parameters described by Yan et al. (2013) [17]. Clean reads from each pig were aligned to the Yorkshire pig reference genome (an unpublished self-assembled genome) using the Burrows–Wheeler Alignment tool (BWA v. 0.7.15) [18] with default parameters. The alignment results were processed using the Picard toolkit (v.1.119, http://broadinstitute.github.io/picard/, accessed on 22 January 2025), to sort the alignments and remove potential PCR duplicates. The processed alignments were indexed using SAMtools (v. 1.6) [19] and further analyzed following the best practices of the Genome Analysis Toolkit (GATK v.4.1.1) [20]. The GenomicsDBImport module was used to merge population-level gVCF files, and genotype calling was performed using GenotypeGVCFs v4.4.0 to generate a population-level VCF file. Variant datasets, including SNPs and InDels, were filtered using hard filtering criteria. Structural variations (SVs) were genotyped using Paragraph (v.2.4a) [21] with default parameters to obtain a population-level SV dataset for a downstream analysis. For the SNP dataset, quality control was performed using PLINK (v.1.9.0) [22]. SNP sites with a minor allele frequency (MAF) < 0.05, genotype call rate < 0.95, individual call rate < 0.95, or Hardy–Weinberg equilibrium (HWE) *p*-value < 0.000001 were excluded. Linkage disequilibrium (LD) pruning was applied using the --indep-pairwise 50 5 0.5 parameter. After filtering, a total of 434,862 SNP sites across 18 autosomes were retained for subsequent genome-wide association study (GWAS) analysis.

### 2.7. Construction of a Map of Intestinal Microbes and Dynamic Changes in Mummium-Fetal Time

To investigate the potential association between the microbial communities in the fecal samples from Yorkshire sows during late pregnancy across six parities and the incidence of mummified fetuses, this study conducted a systematic analysis of microbial functional pathways. A functional enrichment analysis of the microbial communities was performed based on the Gene Ontology (GO) and Kyoto Encyclopedia of Genes and Genomes (KEGG) databases. The statistical significance of the differences between groups was assessed using the pairwise Wilcoxon rank-sum test. To further elucidate the interaction relationships among the microbial taxa, the psych package in R was used to calculate the correlations between the microbial taxa within specific sample types. The Benjamini–Hochberg (BH) method was applied for multiple testing correction of *p*-values. To mitigate the potential bias of zero-inflation effects on co-occurrence network analysis, taxa with a detection rate below 95% in specific sample types were excluded. Prior to calculating the correlation coefficients, the relative abundances of all microbial taxa were log10-transformed. Correlation patterns with Spearman or Pearson correlation coefficient absolute values greater than 0.3 and BH-adjusted *p*-values less than 0.05 were retained. Finally, the microbial interaction network was visualized using Cytoscape (version 3.7.2) [23].

### 2.8. MWAS Analysis

To ensure the reliability of the association analysis, taxa with lower detection rates were excluded, as they provide less informative data. Only taxa present in more than 70% of the samples within a specific sample type were retained for further analysis. The associations between these qualified taxa and the number of mummies (MUM) characteristics were evaluated using a two-part model implemented with a customized R script, as described by Fu et al. (2015) [24]. This model is particularly suitable for microbiome data, as it simultaneously accounts for both binary (presence/absence) and quantitative (abundance) features. The model is expressed as follows:

$$\gamma =\left\{\begin{array}{c} \beta_{1\;}b+e\\ \beta_{2}q+e \end{array}\right.\;$$

where γ is the MUM value, b is a binary feature of a specific microorganism and coded as 0 for absent or l for present for each sample, and q is the log_10_-transformed abundance of a specific microorganism. $\beta_{1}$ and $\beta_{2}$ are the regression coefficients for the binary and quantitative models, respectively, and e is the intercept. The second part of the quantitative analysis was only for the samples in which the specific microorganism was present. The details of the two-part model are illustrated in Figure S1. The *p*-values were obtained from the two-part model association analysis and adjusted by the BH method. If the adjusted *p*-value from the binary model was less than 0.05, the presence or absence of microorganisms could influence the MUM. If the adjusted *p*-value from the quantitative model was less than 0.05, MUM was associated with the relative abundances of the microorganisms.

To identify specific microorganisms significantly associated with MUM, we employed linear discriminant analysis effect size (LEfSe) to detect key microbial species linked to MUM in pigs. Additionally, the Wilcoxon rank-sum test was used to compare the relative abundance of each taxon between the top 40 (highest) and bottom 40 (lowest) pigs ranked by MUM. A microorganism was considered significant only if the adjusted *p*-values from the two-part model association analysis, LEfSe, and Wilcoxon rank-sum test were all less than 0.05. This stringent multi-method approach ensured robust identification of the microbial taxa strongly associated with MUM.

### 2.9. mGWAS Analysis

To identify the microbial species and key genes associated with MUM that are influenced by host genetics, we performed a microbial genome-wide association study (mGWAS) using a linear mixed model implemented in GEMMA (v. 0.98.1). The model is expressed as follows:

γ=Qα+Xβ+g+e

where γ is a vector of corrected phenotypes (the abundance or presence/absence of heritable genera) [25]; Q is a design matrix of covariates, including the top five host genetic principal components calculated [25]; α is a vector of effects for the covariates (including the intercept); X is a vector of allele counts (0, 1, and 2); and β is the SNP effect. g is a vector of polygenic effects that follows the normal distribution *N* (0, G$\sigma_{g}^{2}$), where G is the genetic kinship matrix calculated from genome-wide marker information and $\sigma_{g}^{2}$ is the polygenic additive variance. e is a vector of residual errors. The *p*-values of the SNP effects were calculated using the likelihood ratio test. The Bonferroni correction was used to screen candidate variants, where *p* < 1/*N* represents the potential genome-wide significant threshold, and *p* < 0.05/*N* represents the genome-wide significant threshold. Manhattan plots and QQ plots were generated using the CMplot package in R (v.4.2.0) [26,27].

The reconstruction model described by the extended matrix notation is as follows:

$$\underset{\gamma \in {\mathbb{R}}^{N\times 1}}{\underbrace{\left[\begin{array}{c} y_{1}\\ y_{2}\\ \vdots \\ y_{N} \end{array}\right]}}=\underset{\mathrm{Design}\;\mathrm{matrix}\;Q\in {\mathbb{R}}^{N\times (k+1)}}{\underbrace{\left[\begin{array}{cccc} 1 & Q_{1,1} & \cdots & Q_{1,k}\\ 1 & Q_{2,1} & \cdots & Q_{2,k}\\ \vdots & \vdots & \ddots & \vdots \\ 1 & Q_{N,1} & \cdots & Q_{N,k} \end{array}\right]}}\underset{\alpha \in {\mathbb{R}}^{(k+1)\times 1}}{\underbrace{\left[\begin{array}{c} \alpha_{0}\\ \alpha_{1}\\ \alpha_{k} \end{array}\right]}}+\underset{X\in {\mathbb{R}}^{N\times 1}}{\underbrace{\left[\begin{array}{c} X_{1}\\ X_{2}\\ \vdots \\ X_{N} \end{array}\right]}}\underset{\mathrm{Scalar}}{\underbrace{\beta }}+\underset{g∼N\left(0,G\sigma_{g}^{2}\right)}{\underbrace{\left[\begin{array}{c} g_{1}\\ g_{2}\\ \vdots \\ g_{N} \end{array}\right]}}+\underset{e∼N\left(0,I\sigma_{\varepsilon }^{2}\right)}{\underbrace{\left[\begin{array}{c} e_{1}\\ \begin{array}{l} e_{2}\\ \vdots \end{array}\\ e_{N} \end{array}\right]}}$$

*γ* ∈ R*^N^*^×1^: The corrected phenotype vector, comprising microbial abundance or presence/absence states (after normalization or transformation) for N samples; *Q* ∈ R*^N^*^×(*k*+1)^: The fixed-effect design matrix, including an intercept term (first column of all 1s) and k covariates (e.g., host genetic principal components, batch effects); *α* ∈ R^(*k*+1)×1^: The fixed-effect coefficient vector; *X* ∈ R*^N^*^×1^: the allele count vector of the SNP to be tested, with values of 0 (reference homozygous), 1 (heterozygous), or 2 (variant homozygous); *β* ∈ R: the fixed-effect size of the target SNP, representing the phenotypic contribution per unit allele change; *g* ∈ R*^N^*^×1^: the polygenic random-effect vector, following a multivariate normal distribution; *e* ∈ R*^N^*^×1^: the independent residual vector; I ∈ R^N×N^: the identity matrix; and σ_e_^2^ ∈ R^+^: the residual variance component.

### 2.10. Metabolomics Sequencing Analysis

To investigate the differences in short-chain fatty acid (SCFA) profiles between MUM and non-MUM sows, fecal samples were collected from ten MUM-affected sows and ten unaffected sows of the same parity. Targeted metabolomics sequencing was performed to quantify the SCFA concentrations in the fecal samples. The detailed quantification process is as follows. The SCFA concentrations in the rectal content were measured using a water extraction method (2 volumes of water per weight of sample), followed by protein precipitation with 10% (*v*/*v*) phosphotungstic acid (Sigma-Aldrich, St. Louis, MO, USA), as previously described [28]. For quantification, 2-ethylbutyrate (Sigma-Aldrich) was used as an internal standard. All samples were analyzed in duplicate to ensure accuracy. Data acquisition and peak integration were performed using OpenLab ChemStation C.01.06 software (Agilent, Les Ulis, France). The SCFA concentrations are reported as micromoles per gram of feces (μmol/g feces).

## 3. Results

### 3.1. Frequency Analysis of Mummified Fetuses in Yorkshire Sows Across Different Parities and Dynamic Changes in Gut Microbiota Diversity

**Figure 2:**
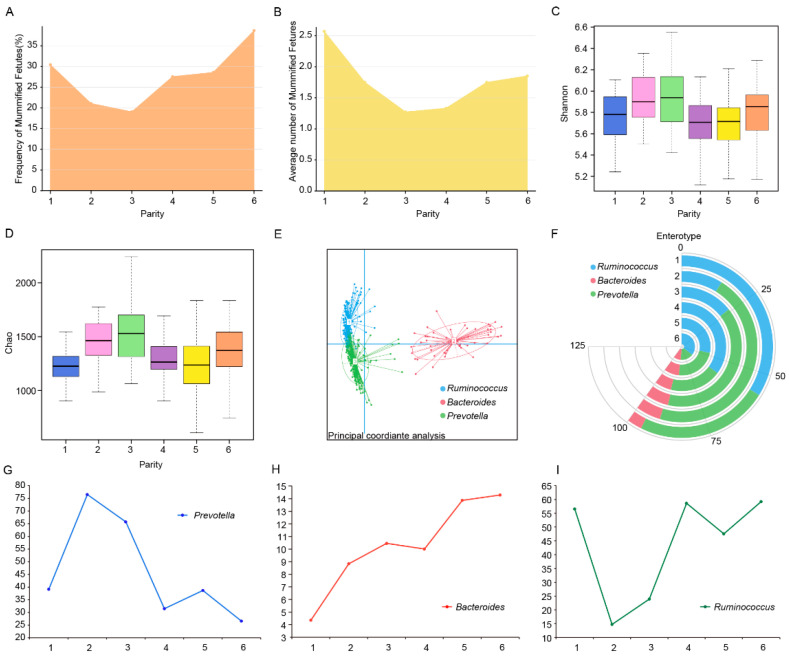
Frequency and number of mummified fetuses and dynamic changes in gut microbiota across parities. (**A**). Dynamic changes in the frequency of mummified fetuses across parities in Yorkshire sows. (**B**). Dynamic changes in the average number of mummified fetuses per individual across parities in Yorkshire sows. (**C**,**D**). Dynamic changes in gut microbiota diversity (Shannon index) and richness (Chao index) across parities. (**E**,**F**). Distribution of enterotypes in the Yorkshire sow population and the composition of enterotypes across parities. (**G**–**I**). Dynamic changes in the proportions of the three enterotypes across parities.

To explore the potential association between the incidence of mummified fetuses and the parity of sows, this study first statistically analyzed the frequency of mummified fetuses in sow populations across different parities. As shown in Figure 2A, the incidence of mummified fetuses reached its lowest value (19.05%) at the third parity, showing an overall trend of first decreasing and then increasing, peaking at the sixth parity (38.68%). To further assess the severity of mummified fetuses, the study calculated the average number of mummified fetuses per affected sow in each parity. The results revealed that the trend in the average number of mummified fetuses was consistent with the incidence: lowest at the third parity (1.27 fetuses) and highest at the first parity (2.57 fetuses) (Figure 2B). To evaluate the potential role of the gut microbiota in the formation of mummified fetuses, the study systematically analyzed the dynamic changes in gut microbial diversity and richness across different parities. The results showed that both microbial diversity and richness reached their highest levels at the third parity (Figure 2C,D), suggesting that the metabolic activity of the gut microbiota in third-parity sows may be at its optimal state, which could be a key factor contributing to the lowest incidence of mummified fetuses. Further analysis of enterotypes in the Yorkshire sow population revealed that their gut microbiota was primarily composed of three enterotypes: *Ruminococcus*, *Bacteroides*, and *Prevotella*. Among these, *Ruminococcus* and *Prevotella* were dominant, but the proportions of enterotypes varied significantly across parities (Figure 2E,F), indicating that the functional characteristics of the gut microbiota in Yorkshire sows are highly parity-dependent. Dynamic analysis showed that the *Prevotella* enterotype peaked in the second and third parities and then gradually declined, while the *Bacteroides* enterotype proportion continued to increase. In contrast, the *Ruminococcus* enterotype exhibited an opposite trend to *Prevotella* (Figure 2G–I). Notably, the trend of the *Prevotella* enterotype was significantly negatively correlated with the incidence of mummified fetuses, while the *Ruminococcus* enterotype trend showed a positive correlation. These results suggest that specific enterotypes of gut microbiota may play a critical role in regulating the formation of mummified fetuses.

### 3.2. Construction of a Dynamic Profile of Gut Microbiota

**Figure 3:**
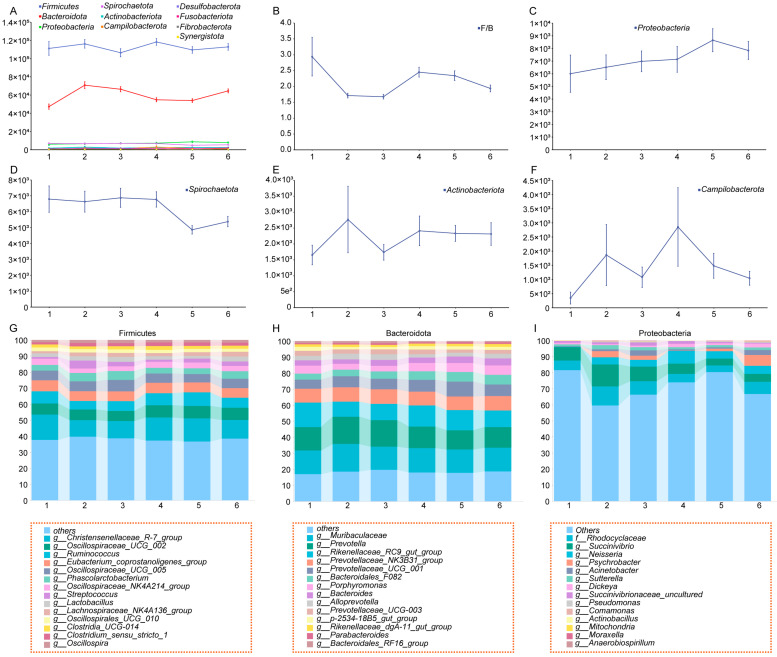
Dynamic changes in gut microbiota at the phylum level. (**A**–**F**). Dynamic changes in the abundance of major phyla in the gut microbiota of Yorkshire sows across parities. (**G**–**I**). Stacked bar plots showing dynamic changes in the abundance of potential probiotics and potential pathogens.

To further investigate the dynamic changes in gut microbiota during gestation, this study constructed dynamic profiles of the gut microbiota at both the phylum and genus levels and identified the core microbial taxa in Yorkshire sows. At the phylum level, microbial community composition analysis revealed that the gut microbiota of Yorkshire sows was primarily composed of *Firmicutes*, *Bacteroidota*, *Proteobacteria*, *Spirochaetota*, *Actinobacteriota*, *Campilobacterota*, and *Desulfobacterota*, regardless of parity (Figure 3A). Among these, *Firmicutes* and *Bacteroidota* accounted for over 80% of the gut microbiota, indicating their dominant roles in the microbial community.

By analyzing the dynamic changes in phylum abundance across parities, we observed significant parity-dependent trends in the major phyla. Specifically, the ratio of *Firmicutes* to *Bacteroidota* (F/B) peaked at parity 1 and gradually decreased, reaching its lowest level at parity 3 (Figure 3B). This suggests that sows in their first parity may experience a state of chronic inflammation due to insufficient physiological adaptation, while their nutrient absorption capacity likely improves with increasing parity. Additionally, the abundance of *Proteobacteria* gradually increased across parities. Notably, *Proteobacteria* includes numerous pathogenic bacteria, and its increased abundance may contribute to the higher incidence of mummified fetuses in later parities. In contrast, the abundance of *Spirochaetota*, *Actinobacteriota*, and *Campilobacterota* exhibited minimal fluctuations, likely influenced more by environmental factors than by parity.

To further elucidate the roles of potential probiotics (*Firmicutes* and *Bacteroidota*) and potential pathogens (*Proteobacteria*) during gestation, we conducted an in-depth analysis of the composition and dynamic changes of the major genera. The results showed that the abundance of major genera exhibited significant parity-dependent variations. Notably, the “Others” category within *Proteobacteria* reached its highest abundance at parity 1, decreased significantly at parity 2, and then gradually increased thereafter (Figure 3G–I). This indicates that the abundance of various minor pathogenic bacteria within *Proteobacteria* increases with parity, which may be a key mechanism underlying the higher incidence of mummified fetuses in later stages of gestation.

**Figure 4:**
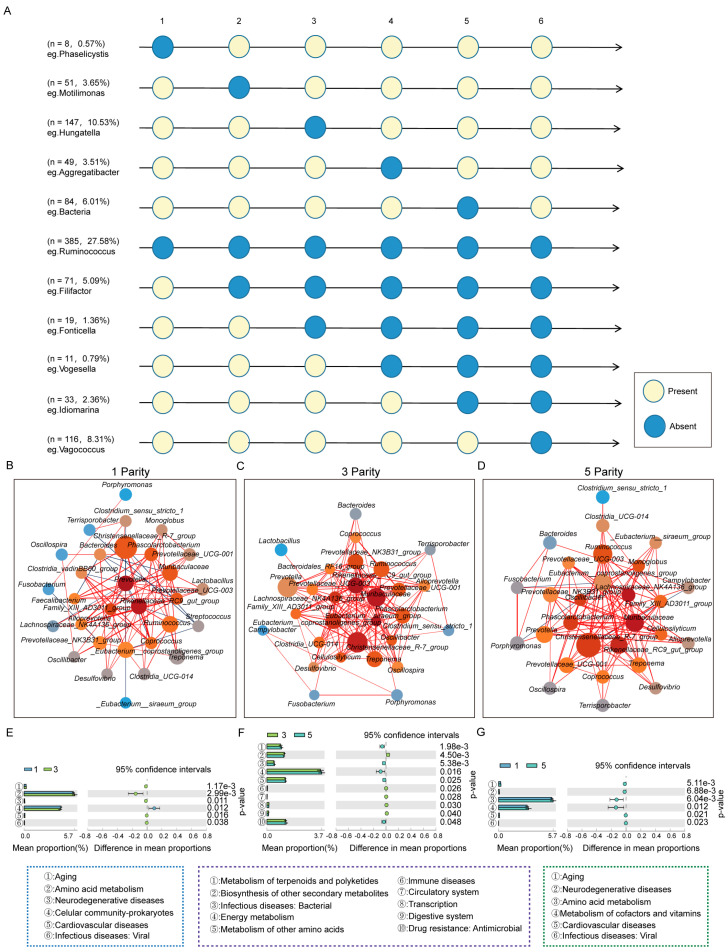
Identification of core gut microbiota and construction of interaction networks and differential functional enrichment analysis across parities in Yorkshire sows. (**A**). Dynamic profiles of gut microbiota and identification of core microbiota. (**B**–**D**). Interaction networks of gut microbiota in Yorkshire sows at the 1st, 3rd, and 5th parities. Co-occurrence network analysis revealed interactions among microbial taxa across parities, where nodes represent microbial taxa and edges represent significant correlations (|r| > 0.3, *p* < 0.05). (**E**–**G**). Differential functional enrichment analysis of gut microbiota across parities in Yorkshire sows. Note: In the interaction networks, node color represents the core level of the microbiota, with darker red indicating a higher core level; node size reflects relative abundance, with larger nodes indicating higher abundance; the color of the connections between nodes indicates the correlation, with red indicating positive correlation and blue indicating negative correlation.

Given that gut microbiota may function independently at specific parities or play a continuous role throughout the sow’s reproductive cycle, this study systematically constructed a dynamic profile of genus-level microbiota in Yorkshire sows across parities. As shown in Figure 4A, the study identified eight microbial taxa unique to first-parity sows, 51 taxa in the second parity, 147 taxa in the third parity, 49 taxa in the fourth parity, 84 taxa in the fifth parity, and 116 taxa in the sixth parity. Additionally, the study identified 385 core microbial taxa that persisted throughout the reproductive cycle, indicating their essential physiological roles across all parities. Furthermore, 71 taxa persisted after the second parity, 19 after the third parity, 11 after the fourth parity, and 33 taxa were unique to the fifth and sixth parities (Figure 4A, Table S2).

To further elucidate the interactions of the gut microbiota across parities, this study constructed corresponding gut microbial ecological networks. The analysis revealed significant differences in the microbial networks among parities. In first-parity sows, *Prevotella*, *Rikenellaceae_RC9_gut_group*, and *Family_XIII_AD3011_group* were identified as core taxa, with core ranks greater than 10, suggesting that these taxa not only function independently but may also regulate the abundance of other taxa to collectively participate in physiological regulation. In third-parity sows, 15 core taxa with ranks greater than 10 were identified, including the *Rikenellaceae_RC9_gut_group*, *Muribaculaceae*, *Eubacterium__siraeum_group*, *Christensenellaceae_R7_group*, *Eubacterium__coprostanoligenes_group*, *Lachnospiraceae_NK4A136_group*, *Prevotellaceae_NK3B31_group*, *Ruminococcus*, *Prevotellaceae_UCG-001*, *Phascolarctobacterium*, *Oscillibacter*, *Treponema*, *Cellulosilyticum*, *Clostridia_UCG-014*, and *Family_XIII_AD3011_group*. This indicates that the gut microbial ecosystem in third-parity sows is more diverse and well-developed compared to first-parity sows. However, in fifth-parity sows, the number of core taxa decreased to nine, including the *Eubacterium__coprostanoligenes_group*, *Lachnospiraceae_NK4A136_group*, *Muribaculaceae*, *Rikenellaceae_RC9_gut_group*, *Christensenellaceae_R-7_group*, *Phascolarctobacterium*, *Oscillibacter*, *Ruminococcus*, and *Monoglobus*. This suggests that, after reaching the most developed microbial interaction network in the third parity, the diversity of core taxa gradually declines with increasing parity, which may be one of the underlying causes of the rising incidence of mummified fetuses (Figure 4B–D).

To further validate the impact of the dynamic changes in gut microbiota on physiological functions, this study conducted a functional enrichment analysis of the gut microbiota across parities. The results showed that, compared to first-parity sows, the gut microbiota of third-parity sows exhibited significant downregulation in the pathways related to amino acid metabolism, cardiovascular diseases, and neurodegenerative diseases, which may explain the lower incidence of mummified fetuses. Additionally, compared to fifth-parity sows, the gut microbiota of third-parity sows showed significant upregulation in the pathways related to the biosynthesis of other secondary metabolites, immune diseases, the circulatory system, transcription, and the digestive system, while downregulating pathways associated with the metabolism of terpenoids and polyketides and bacterial infectious diseases. These functional differences further support the critical role of dynamic changes in the gut microbiota during sow gestation (Figure 4E–G).

### 3.3. Identification of Microbiota Markers Significantly Associated with MUM Through Multi-Model MWAS

**Figure 5:**
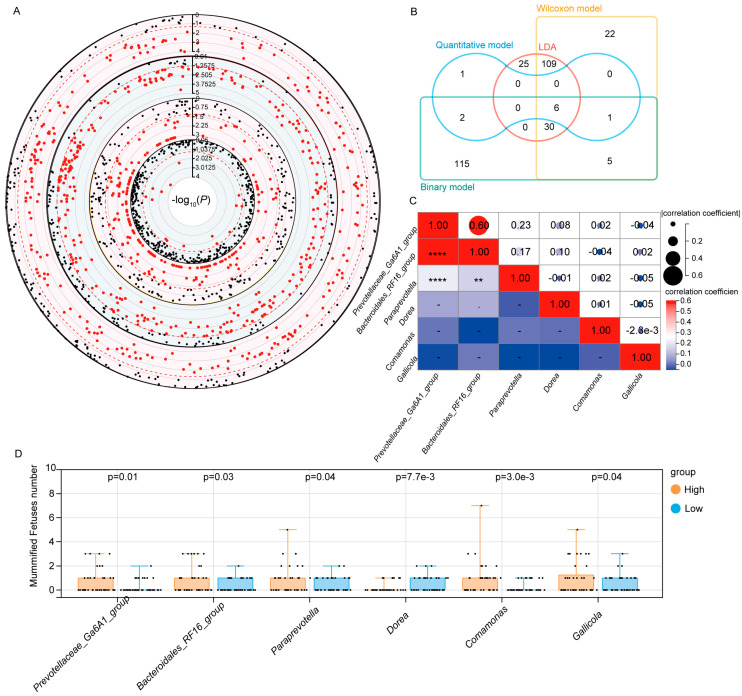
MWAS analysis and meta-analysis results. (**A**,**B**). Results of MWAS analysis, meta-analysis, and Venn diagram. (**C**). Heat map of correlations among key gut microbes. (**D**). Relationship between key microbiota and phenotype. Note: In (**A**), the red dots represent microbial taxa significantly associated with MUM across different models, while the black dots indicate taxa with no significant association to MUM. In (**C**), asterisks (*) denote correlation strength: ** *p* < 0.05 (significant), **** *p* < 0.001 (strongest significance).

To further pinpoint the key microbiota associated with MUM, this study conducted MWAS using multiple models. As shown in Figure 5A, a linear discriminant analysis (LDA) identified 171 microbial taxa, the Wilcoxon model identified 173 taxa, the binary model identified 160 taxa, and the quantitative model identified 10 taxa associated with MUM. Through a meta-analysis of all model results, we ultimately identified six microbial taxa that were significantly associated with MUM across all models, including *Bacteroidales_RF16_group*, *Prevotellaceae_Ga6A1_group*, *Comamonas*, *Paraprevotella*, *Dorea*, and *Gallicola* (Figure 5B). To evaluate the interactions among these key microbial taxa, we further analyzed their correlation network. The results revealed a strong positive correlation between the *Bacteroidales_RF16_group* and the *Prevotellaceae_Ga6A1_group*, with a correlation coefficient as high as 0.6. To validate these results, we employed a two-tailed distribution method, selecting the 40 sows with the highest and lowest abundances of each identified taxon, and statistically analyzed their MUM counts. The results showed that the *Bacteroidales_RF16_group*, *Prevotellaceae_Ga6A1_group*, *Comamonas*, *Paraprevotella*, and *Gallicola* were significantly positively correlated with the incidence of mummified fetuses, meaning that, as the abundance of these taxa increased, so did the incidence of MUM. Notably, *Dorea* exhibited a significant negative correlation with the incidence of mummified fetuses, suggesting that it may play a protective role in reducing MUM incidence by regulating gut microbial balance (Figure 5D and Figure S2).

### 3.4. Association Between Host Genetics and the Key Microbiota Related to MUM

**Figure 6:**
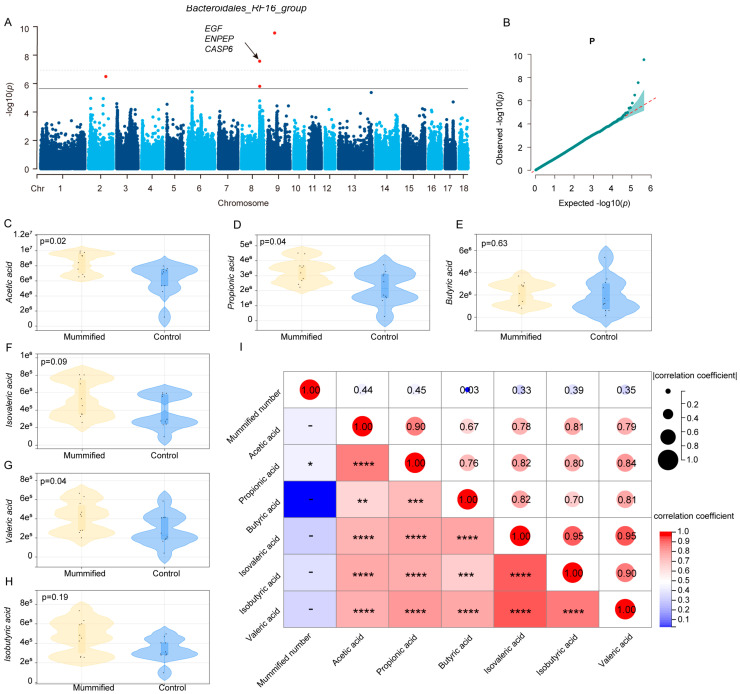
mGWAS analysis and metabolomic differential analysis. (**A**,**B**). Manhattan plot and QQ plot of mGWAS analysis for *Bacteroidales_RF16_group*. (**C**–**H**). Differential analysis of key metabolites in feces between sows with MUM and normal sows. (**I**). Heatmap of correlations between mummified fetuses and SCFAs. Note: The asterisks (*) indicate the strength of correlations: *: Correlation exists but does not reach significance; **: Significant correlation (*p* < 0.05); ***: Highly significant correlation (*p* < 0.01); ****: Strongest correlation (*p* < 0.001).

To further investigate the roles of host genetics and metabolites in the process of MUM formation influenced by gut microbiota, we conducted mGWAS on the six key microbial taxa associated with MUM. The results are shown in Figure 6, revealing that only the *Bacteroidales_RF16_group* is regulated by host genetics. Specifically, we identified one significant locus (rs695637165) on chromosome 2, two significant loci (rs345237235 and rs3475666995) on chromosome 8, and one significant locus (rs703398857) on chromosome 9. Additionally, we screened candidate genes such as *EGF*, *ENPEP*, and *CASP6* (Figure 6A,B; Table S4). These findings suggest that host genetic factors may indirectly influence the formation of mummified fetuses by regulating the abundance of the *Bacteroidales_RF16_group*. On the other hand, we analyzed the SCFA content in the feces of sows with mummified fetuses and normal sows. The results showed that the levels of multiple SCFAs, including acetic acid, propionic acid, butyric acid, and isobutyric acid, were significantly higher in sows with mummified fetuses compared to normal sows. Among these, the differences in acetic acid, propionic acid, and valeric acid were particularly significant (*p* < 0.05), suggesting that these three SCFAs may play an important role in the formation of mummified fetuses (Figure 6C–H). To further validate the association between SCFAs and mummified fetuses, we analyzed the correlation between the levels of various SCFAs and the number of mummified fetuses. The results indicate that the levels of multiple SCFAs correlate with the number of mummified fetuses, with the strongest correlations observed for acetic (r = 0.44) and propionic (r = 0.45). These results further support the potential role of SCFAs in the formation of mummified fetuses and provide important metabolite targets for subsequent research (Figure 6I).

## 4. Discussion

The relationship between sow parity and reproductive success is a complex and multifaceted issue, and its impact on fetal growth and development has long been a critical topic in reproductive biology research. Over the past three decades, researchers have focused on exploring the mechanisms linking parity with pregnancy success rates and the occurrence of MUM [29]. Studies have shown that host genetic variation is a core factor driving phenotypic variation, and emerging evidence in recent years has further revealed the potential role of gut microbiota heterogeneity in individual phenotypic differences [30,31]. As the “second genome” of the host [32], the gut microbiome plays a pivotal regulatory role in various physiological processes, including disease immunity, nutrient metabolism, growth, and development [33,34]. However, the specific mechanisms by which host genetics shape the structure of the gut microbiota and how the two synergistically influence fetal development during pregnancy to ultimately determine pregnancy outcomes remain largely unknown. Deep whole-genome sequencing at 10× coverage was performed for host genomic analysis, while 16S rRNA gene sequencing was used to systematically characterize the gut microbiome. Additionally, a metabolomic analysis was conducted on individuals exhibiting significant mummified fetus variation to comprehensively reveal the underlying molecular mechanisms. This study achieved innovative breakthroughs in the following aspects. First, we constructed, for the first time, an association map between the dynamic changes in the gut microbiota and the incidence of mummified fetuses in sows of different parities. Through a quantitative analysis, we identified correlations between the abundance of specific microbial taxa and the occurrence of mummified fetuses, providing reliable evidence for identifying the key microbiota influencing this phenomenon. Second, within the framework of gut microbiota research, we innovatively integrated statistical models and methodologies from quantitative genetics. This not only deepened our understanding of the interaction mechanisms between the host genetics and gut microbiota but also systematically evaluated the effects of microbial communities on host mummified fetus phenotypes. Finally, through metabolomic analysis, we elucidated the mechanisms by which host genetics and gut microbiota interactions influence mummified fetus phenotypes via metabolic pathways, offering new molecular biological perspectives on sow reproductive health. By employing a multi-omics integration analysis strategy, this study not only expanded our understanding of the mechanisms underlying sow reproductive biology but also provided important theoretical foundations and practical guidance for developing reproductive management strategies based on gut microbiota modulation. The findings have significant application value for improving sow reproductive efficiency and optimizing breeding management.

In constructing the dynamic atlas of the gut microbiota in Yorkshire sows, we systematically observed the periodic changes in microbial diversity and richness (α-diversity) across parities 1 to 6. Notably, the third parity exhibited the most diverse microbiota, which may be associated with the presence of abundant probiotics. These probiotics may regulate the physiological functions of pregnant sows, thereby reducing the incidence of mummified fetuses [35]. The study found that microbial richness gradually increased during the transition from the first to the third parity, significantly declined in the fourth parity, and then showed an increasing trend from the fifth to the sixth parity. This finding aligns with previous research [36,37], further confirming the dynamic regulatory role of the gut microbiota during the host’s physiological cycle. Similar dynamic patterns of microbial community changes have been reported in human studies, where the gut microbiome undergoes significant transformations from infancy to adulthood under the influence of diet, medication, and physiological needs [10]. Through a systematic analysis of the enterotype dynamics and phylum-level microbial characteristics in Yorkshire sows, we further validated these observations. The study revealed that enterotypes driven by *Ruminococcus*, *Bacteroides*, and *Prevotella* exhibited significant differences in microbial composition, metabolic functions, and health impacts. Specifically, the *Ruminococcus*-driven enterotype was associated with enhanced nutrient absorption and metabolic efficiency, while the *Prevotella*-driven enterotype was closely linked to improved metabolic health [38,39]. In this study, regardless of parity, the Yorkshire sow population was primarily divided into three enterotypes. During the second and third parities, the *Prevotella*-driven enterotype dominated, indicating a significant enhancement in the sows’ ability to absorb nutrients through the gut microbiota. In contrast, the *Ruminococcus*-driven enterotype gradually became dominant in the first parity and subsequent parities after the third, reflecting not only improved nutrient absorption but also suggesting the possible onset of subtle chronic inflammatory responses in the sow’s gut. These findings are highly consistent with the functional characteristics of the respective enterotypes [39,40]. At the phylum level, we observed a certain degree of decline in the Firmicutes-to-Bacteroidetes ratio (F/B ratio) between the first and second parities. This change reflects the maturation process of individual sow development, with nutrient absorption primarily shifting to support successful pregnancy [41,42]. On the other hand, the abundance of Proteobacteria gradually increased from the first parity. Notably, Proteobacteria include several potential pathogens, such as *Escherichia coli*, *Salmonella* spp., and *Shigella* spp. These pathogens can cause severe consequences, including piglet diarrhea, growth retardation, and reduced feed efficiency, through direct infection or environmental contamination [43,44]. Based on these findings, we propose that the period following the third parity is a critical window for implementing exogenous suppression of pathogenic bacteria. This discovery provides important theoretical foundations and practical guidance for optimizing sow reproductive management strategies.

In constructing the dynamic atlas of the gut microbiota at the genus level in Yorkshire sows, we systematically identified the core microbiota and microbial taxa that play specific roles during particular physiological stages. By definition, the core microbiota refers to the bacterial taxa present in at least one sow at all time points [1]. Among the 1396 genera analyzed, we identified 385 microbial taxa as the core microbiota throughout the production cycle of Yorkshire sows. Additionally, we identified 71 genera that significantly increased after the first parity, indicating adaptive changes in the gut microbiota to meet the specific physiological needs of the sows. These findings are consistent with previous research [45], further revealing the dynamic regulatory role of the gut microbiota during the host’s physiological cycle.

Next, we focused on the 385 core microbial taxa and employed a two-part model, rank-sum test, and linear discriminant analysis (LDA) to investigate the microbiota associated with sow MUM. The binary component of the two-part model was used to analyze the presence or absence of microbiota, thus utilizing all 1396 genera for comprehensive analysis. The results showed that microbial taxa such as *Bacteroidales_RF16_group*, *Prevotellaceae_Ga6A1_group*, *Comamonas*, *Paraprevotella*, *Dorea*, and *Gallicola* were significantly associated with host MUM. These findings align closely with previous research. For example, the *Bacteroidales_RF16_group* positively influences sow pregnancy outcomes by improving serum metabolites [46]. The *Prevotellaceae_Ga6A1_group* alleviates fetal growth restriction in pregnant rats by inhibiting the TLR9/MYD88 pathway [47], and *Dorea*, as a potentially harmful bacterium, may negatively impact the health of pregnant sows [48]. Although the aforementioned studies provide partial support for the findings of this research, existing studies primarily focus on the potential effects of key microbial taxa on sow pregnancy outcomes, with less attention given to the direct relationship between the gut microbiota and MUM. Moreover, current research predominantly emphasizes the influence of environmental or dietary factors on microbiota and their indirect effects on host phenotypes, while studies on the role of host genetics in driving gut microbiota changes and their impact on reproductive performance remain limited. Given this research gap, we further explored the genetic mechanisms of gut microbiota–host interactions and their effects on reproductive performance, providing new perspectives and theoretical foundations for understanding the microbial regulation of sow reproductive performance.

In this study, we employed the mGWAS approach to investigate the regulatory mechanisms of host genetics on the gut microbiome, focusing on the abundance of six key microbial taxa significantly associated with the MUM phenotype in Yorkshire sows. The results revealed that the abundance of the Bacteroidales_RF16_group is significantly influenced by host genetic factors. Furthermore, we identified several candidate genes (e.g., EGF, ENPEP, and CASP6) and their associated SNP loci that are linked to the abundance of key microbial taxa. These findings provide novel insights into the molecular mechanisms by which host genetics shapes the composition of the gut microbiome. EGF, one of the primary ligands for the epidermal growth factor receptor (EGFR), triggers EGFR dimerization through ligand–receptor binding, thereby activating the intracellular tyrosine kinase (TK) domain. This process involves the conversion of ATP to ADP and the phosphorylation of TK, ultimately leading to the activation of EGFR enzymatic activity. Activated EGFR transmits proliferative and anti-apoptotic signals to the nucleus through downstream signaling pathways, such as PI3K-AKT-mTOR and Ras-Raf-MEK-ERK1/2, regulating cellular growth and division [49,50,51]. EGF plays a critical role in embryonic development, placental formation, and uterine repair. Although there is no direct evidence linking this gene to the occurrence of mummified fetuses in pigs, it may indirectly influence reproductive efficiency by regulating endometrial repair or embryo implantation processes. The ENPEP gene encodes glutamyl aminopeptidase, an enzyme that plays a significant role in peptide metabolism and immune regulation. Studies have shown that ENPEP is crucial for embryonic formation and placental development, and its abnormal expression in the placenta may be associated with pregnancy-related disorders [52,53]. Therefore, ENPEP can be considered a potential candidate gene that indirectly regulates the occurrence of mummified fetuses in sows. Mummified fetuses refer to an abnormal phenomenon where a fetus dies during gestation but is not expelled by the mother, leading to dehydration and calcification within the uterus. The CASP6 gene is involved in apoptosis and inflammatory responses, particularly during embryonic development and programmed cell death. Given its functional characteristics, CASP6 is also regarded as a potential candidate gene influencing the frequency of mummified fetuses in sows [54,55]. These findings provide critical clues for understanding the molecular mechanisms underlying the formation of mummified fetuses in sows and lay a theoretical foundation for subsequent functional validation and breeding applications.

The gut microbiota significantly influences host reproductive performance through multiple metabolic pathways. The sow reproductive cycle is tightly regulated by hormones, with luteinizing hormone (LH) and follicle-stimulating hormone (FSH) serving as key regulators. LH induces the development of pre-ovulatory follicles, triggers ovulation, and promotes corpus luteum formation and progesterone (P) secretion. FSH synergizes with LH to regulate estradiol (E_2_) secretion. From early to late gestation, the plasma levels of prolactin (PRL), progesterone (P), FSH, and estrogen gradually decline. Estradiol and progesterone play pivotal roles in endometrial gland development, with estradiol driving the proliferation of uterine luminal and glandular epithelium, creating an optimal environment for embryo implantation and development [56,57,58]. Beyond hormonal regulation, the gut microbiota impacts sow reproductive performance through the production of short-chain fatty acids (SCFAs), including acetate, propionate, and butyrate, derived from the fermentation of dietary fiber and resistant starch. These SCFAs regulate energy homeostasis and are crucial for maintaining intestinal homeostasis, modulating immune responses, and exerting anti-inflammatory effects [59,60]. Short-chain fatty acids (SCFAs, e.g., acetate) influence fetal development through the maternal metabolism. As a primary energy substrate, acetate can cross the placental barrier to promote fetal lipid synthesis and organ growth, while simultaneously modulating maternal gut microbiota homeostasis and enhancing placental angiogenesis and nutrient transport. Studies demonstrate a positive correlation between maternal acetate levels and both fetal neurodevelopment and metabolic health. However, excessive acetate may disrupt epigenetic regulation, highlighting the need for balanced dietary fiber intake to optimize maternal–fetal benefits [59]. A metabolomic analysis in this study revealed significant alterations in the SCFA profiles within the intestinal tracts of sows carrying mummified fetuses. Key metabolites, including acetate, propionate, and valerate, showed markedly elevated levels compared to the healthy sow group (*p* < 0.05). A subsequent correlation analysis demonstrated that abnormal accumulation of these SCFAs may be closely associated with the pathological progression of fetal mummification, with particularly prominent concentration changes observed in acetate, propionate, and valerate. These findings suggest that these metabolites may impair normal fetal development by modulating the immune microenvironment at the maternal–fetal interface and/or inducing energy metabolism dysregulation. This discovery provides novel insights into the metabolic regulation mechanisms underlying pregnancy disorders [61]. Our findings demonstrate that host genes modulate the abundance of gut microbiota, which in turn, regulates microbial metabolites, ultimately impacting reproductive outcomes. This study provides additional evidence supporting the intricate interplay between host genetics, gut microbial composition, and metabolic activity in determining pregnancy success.

## 5. Conclusions

This study constructed a comprehensive dynamic atlas of the gut microbiota in Yorkshire sows across different parities, systematically identifying critical windows for probiotic supplementation and the suppression of sow-derived pathogens. Additionally, we characterized the core microbiota that consistently functions in the sow’s gut. Using MWAS and meta-analysis, we identified key microbial communities closely associated with MUM. Furthermore, mGWAS elucidated the genetic basis underlying these microbial communities. Innovatively integrating multi-omics analysis, this study explored the intricate regulatory network of “host genetics—gut microbiota—host phenotype”. We identified microbial communities significantly linked to genomic variations, as well as multiple candidate genes and significant SNP loci. This work enriches the genetic information database related to pig MUM and provides a scientific foundation for enhancing sow reproductive performance and utilization efficiency—traits of substantial economic importance.

## Data Availability

The original contributions presented in this study are included in the article. Further inquiries can be directed to the corresponding authors.

## Supplementary Materials

The following supporting information can be downloaded at: https://www.mdpi.com/article/10.3390/microorganisms13051052/s1, Figure S1: Principle schematic of the two-part model; Figure S2: Heatmap of correlations between MUM and key microbial taxa; Table S1: Composition and nutritional levels of the base diet; Table S2: Analysis of dynamic changes in microbial composition across sow parity; Table S3: The key microbial taxa associated with MUM identified through MWAS analysis across different models; Table S4: The key SNP loci with the smallest *p*-values identified through mGWAS analysis.
