# Supplementary material for: Host–Microbiota Interactions in the Pathogenesis of Porcine Fetal Mummification
Source: Microorganisms. 2025 Apr 30;13(5):1052. doi: 10.3390/microorganisms13051052 (PMC12113762; doi:10.3390/microorganisms13051052)
Supplement: Supplementary file 1 [file microorganisms-13-01052-s001.zip › Supplementary File 1 Table S1.pdf]

Table S1. Composition and nutritional levels of the base diet.

Diet Composition	Content /%	Level of Nutrition	Content/%
Corn	63.10	Metabolizable Energy/MJ·kg^{-1}	14.23
Soybean meal (43%)	25	Crude protein	17.8
Bran	4	Total lysine	1.03
Choice white grease	2	Ca	0.71
Fish meal (67%)	2	Total phosphorus	0.56
Sow Vit-Min premix	0.50	Effective phosphorus	0.34
Salt	0.40		
Dicalcium phosphate	0.90		
Limestone	0.80		
Lysine	0.15		
Methionine	0.05		
Threonine	0.03		
Tryptophan	0.02		
Choline chloride	0.1		
Zeolite powder	0.95		
Total amount	100		

Note: The premix provides, per kilogram of feed, vitamin A, 10,000 IU; vitamin D3, 1800 IU; vitamin E, 100 IU; vitamin K3, 4.5 mg; vitamin B1, 2.0 mg; riboflavin, 6.0 mg; vitamin B6, 7.0 mg; vitamin B12, 0.05 mg; niacin, 30 mg; pantothenic acid, 35 mg; folic acid, 3.5 mg; biotin, 0.5 mg; choline chloride, 500 mg; iron, 80 mg; copper, 20 mg; zinc, 100 mg; manganese, 25 mg; iodine, 0.14 mg; and selenium, 0.15 mg.
