# Supplementary material for: Host–Microbiota Interactions in the Pathogenesis of Porcine Fetal Mummification
Source: Microorganisms. 2025 Apr 30;13(5):1052. doi: 10.3390/microorganisms13051052 (PMC12113762; doi:10.3390/microorganisms13051052)
Supplement: Supplementary file 1 [file microorganisms-13-01052-s001.zip › Supplementary File 6 Table S4.pdf]

Table S4 The information of the SNP with the most significant p-value in the mGWAS results.

chr	pos	*P*
8	112600340	0.000127538
15	139214785	0.000233659
7	7815962	0.000251147
2	17353167	0.000262637
18	13631641	0.000309458
2	21611233	0.000316468
3	2522669	0.000347709
8	113411488	0.00037773
14	124796461	0.000384674
