# Supplementary figures and images for: Host–Microbiota Interactions in the Pathogenesis of Porcine Fetal Mummification
Source: Microorganisms. 2025 Apr 30;13(5):1052. doi: 10.3390/microorganisms13051052 (PMC12113762; doi:10.3390/microorganisms13051052)

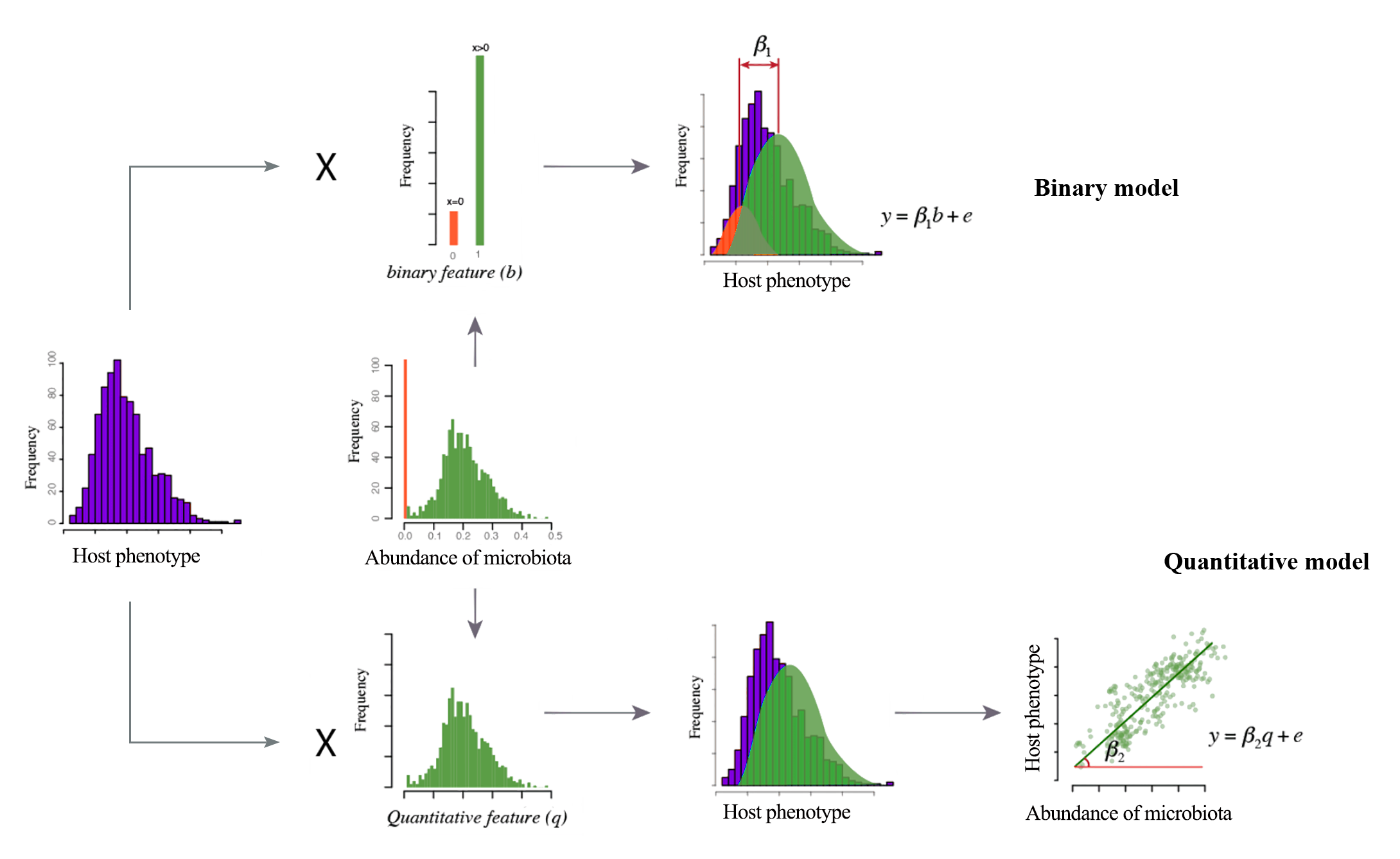

Supplement: Supplementary file 1 [file microorganisms-13-01052-s001.zip › Supplementary File 2 Figure S1.tif]

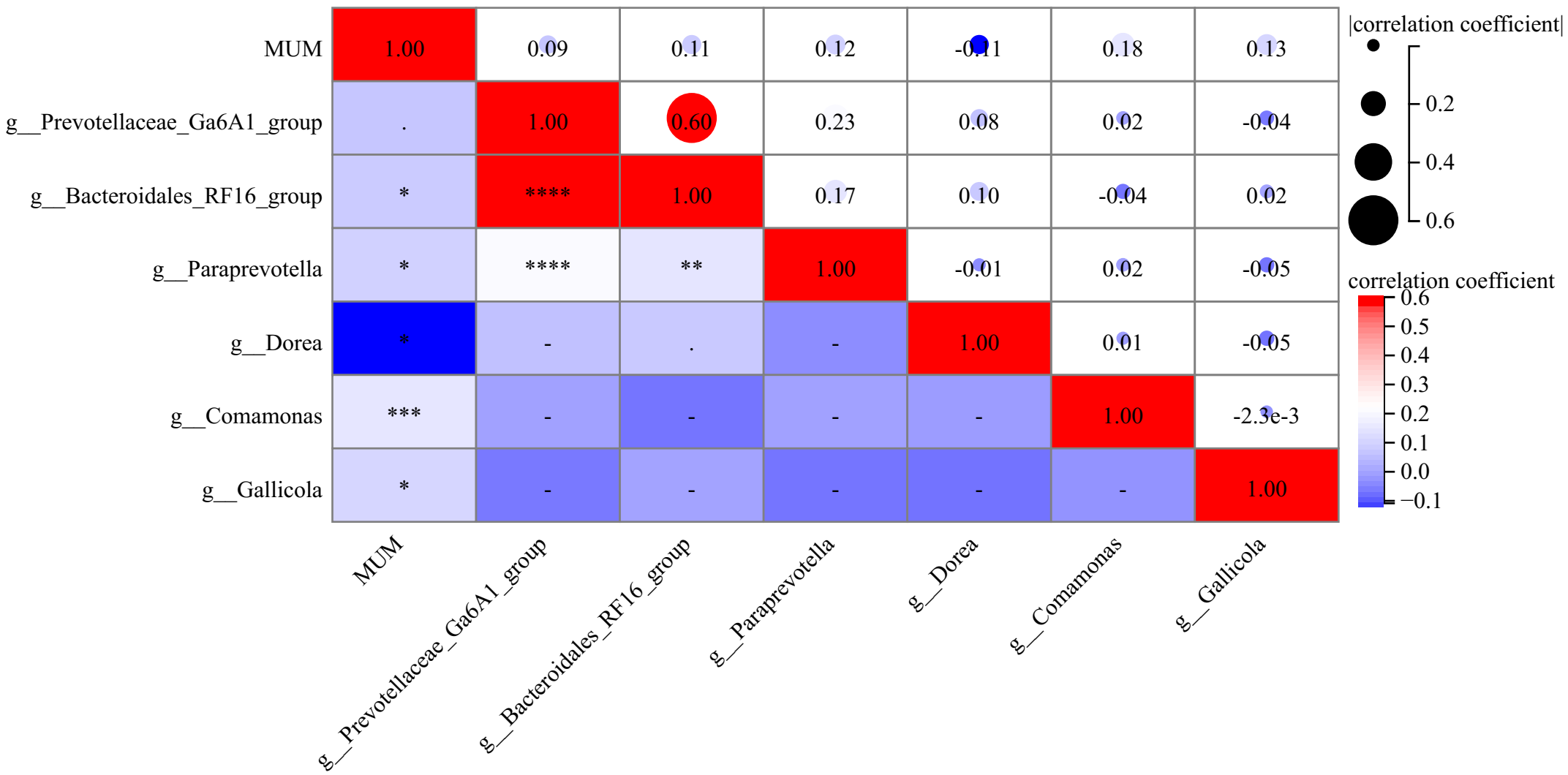

Supplement: Supplementary file 1 [file microorganisms-13-01052-s001.zip › Supplementary File 5 Figure S2.pdf]
